# SOCIAL ISOLATION AND LONELINESS: A LATENT CLASS APPROACH TO COSTING HEALTH SERVICE ENGAGEMENT IN AGING POPULATIONS

**DOI:** 10.1093/geroni/igz038.1967

**Published:** 2019-11-08

**Authors:** Elaine Douglas, David Bell

**Affiliations:** 1 University of Stirling, Stirling, Scotland, United Kingdom; 2 University of Stirling, Stirling, United Kingdom

## Abstract

Social isolation and loneliness are associated with poorer health status and poorer health outcomes. Little is known the impact on health service usage, and its inherent cost, although it is considered to be higher. Latent class analysis (LCA) was used to determine profiles (population groups) of loneliness and social isolation in older people (aged 50+, n=1,057) using model-fit criteria. Loneliness was measured using the UCLA Loneliness Scale and social isolation used a measure of social networks and social contact. We then analysed the socio-demographic, perceived health, and health behaviour of these profiles using descriptive statistics and logistic regression. The survey data (HAGIS, 2016/17) were linked to retrospective administrative health data to investigate patterns of repeat prescription use (from 2009) and health service usage (from 2005) and their associated costs. Our results highlight the distinction and inter-relation between social isolation and loneliness (including associations with socio-demographic and health characteristics), and the variation in health service usage and costs between the population groups. LCA profiles may help focussed targeting of these groups for health interventions. Further, the data-driven approach of LCA may overcome some of the limitations of indices of social isolation and loneliness. As such, this will extend the existing methodological approaches to quantitative analyses of social isolation and loneliness and demonstrate the benefits of using linked administrative health data. Significantly, this study incorporates the social and financial cost of social isolation and loneliness on health and its implications for health services.

